# New Challenges, Evolved Approach: The Public Health Response Readiness Framework

**DOI:** 10.1089/hs.2023.0056

**Published:** 2023-09-19

**Authors:** Kate C. Noelte, Christine Kosmos, Amanda McWhorter

**Affiliations:** Division of State and Local Readiness, Office of Readiness and Response, US Centers for Disease Control and Prevention, Atlanta, GA.

**Keywords:** Public health preparedness/response, COVID-19, Public Health Emergency Preparedness

## Introduction

Since its inception following the events of September 11, 2001, the US Centers for Disease Control and Prevention (CDC) Division of State and Local Readiness (DSLR) has supported the development and sustainability of response-ready state, tribal, local, and territorial (STLT) public health departments through the Public Health Emergency Preparedness (PHEP) program.^1,2^ The program’s cooperative agreements provide guidance, funding, field staff, technical assistance, and resources to 62 response-ready public health departments across the nation. The program is grounded in the 15 public health emergency preparedness and response capabilities, outlined in *Public Health Emergency Preparedness and Response Capabilities: National Standards for State, Local Tribal, and Territorial Public Health*,^3^ which serve as national standards for public health readiness and provide a blueprint that guides the development of STLT preparedness programs nationwide.

Recipients of the cooperative agreements have credited these preparedness and response capabilities and the dedicated PHEP program funding with creating a strong foundation that readies jurisdictions for all types of public health emergencies, from localized events, such as small-scale disease outbreaks, weather-related events, and environmental hazards, to large-scale catastrophic events such as global pandemics.^4^ The scale, scope, and complexity of the COVID-19 pandemic challenged even the most prepared jurisdictions.

As made evident during simultaneous COVID-19 pandemic, mpox (monkeypox) outbreaks, and severe hazard events such as Hurricane Ian, public health emergency preparedness is constantly changing and growing more complex. DSLR recognized the need to adapt and meet the challenges of future public health threats, including the increasing demand placed on STLT programs due to back-to-back response operations, and began to envision the next generation of the PHEP program. The demands of the COVID-19 pandemic alone solidified the need for guidance to address complex emergencies, as STLT public health preparedness programs reported that most of their efforts were directed toward response activities and that routine preparedness efforts were often suspended during constant response operations. The PHEP program encouraged further development of the CDC public health preparedness capability model to support the development of specific capabilities that build strong public health response capacity.

This commentary describes the development of the new Public Health Response Readiness Framework that transforms 2 decades of progress, best practices, and lessons learned during multiple public health responses into a model for advancing public health response. The aim of the framework is to guide the work of CDC and STLT public health departments and direct the focus and resources toward the most critical components of response readiness. Grounded in the 15 preparedness capabilities,^3^ the framework focuses on 10 essential priorities for the future of response (Figure), which help determine what specific response actions are needed to ensure response readiness in the future. Following the development of the framework, a work group for each essential priority was convened to identify or develop preparedness, response, and recovery actions for DSLR and CDC, the 62 PHEP program jurisdictions, and national partners.

## Framework Impetus and Inputs

Spurred by insights raised during the COVID-19 pandemic and other recent responses, as well as 20 years of program experience,^5^ DSLR embarked on an extensive program review to evaluate the effectiveness of the PHEP program for ensuring response readiness for 21st century threats.^6^ The timing of this effort aligned well with the upcoming PHEP program funding cycle and allowed DSLR the opportunity to implement a fresh approach to readiness.^7,8^ To inform the strategy, DSLR compiled inputs from a wide array of partners and collaborators (Box).

DSLR provided funding to Association of State and Territorial Health Officials (ASTHO) and the National Association of County and City Health Officials (NACCHO) to solicit formal feedback from their members. National organizations compiled more than 200 topic areas proposed by STLT partners (ASTHO, unpublished data).^6,9^ DSLR gathered input from its routine engagement with members of ASTHO’s Directors of Public Health Preparedness Executive Committee, leaders from ASTHO, NACCHO, the Council of State and Territorial Epidemiologists, and the Association of Public Health Laboratories. The CDC Office of Readiness and Response and other offices across the agency also provided extensive feedback. Finally, the framework’s strategy was informed by PHEP Operational Readiness Review data submitted to DSLR by recipients as a requirement of the PHEP Cooperative Agreement, which identified jurisdictions’ readiness strengths and gaps.^10^ This feedback was analyzed and the themes were categorized into the 10 priority areas that form the basis of the framework.

The Public Health Response Readiness Framework is a tool to support public health readiness, from preparedness planning to response and recovery. Many priorities identified in the framework represent enhancements of the existing 15 preparedness capabilities and areas that are most critical for ensuring response-ready STLT public health departments. The PHEP program is transitioning from building capabilities to applying capabilities across these 10 essential priority areas of response readiness. The preparedness and response capabilities identify all the elements needed to build successful preparedness programs at STLT levels, while the response framework identifies the critical factors that must be in place to successfully execute a response to public health threats.

DSLR intends to use the framework to drive future public health emergency preparedness and response work. Specifically, it is using the framework priorities to (1) shape requirements and guidance for the 2024-2028 PHEP notice of funding opportunity, (2) steer DSLR’s PHEP activities in support of STLT public health departments, and (3) inform DSLR’s guidance to nongovernmental organizations (NGOs) in support of STLT public health departments.

## Public Health Response Readiness Framework

The framework outlines 10 priority areas that are essential to public health response readiness moving forward. In this section, each of these priority areas are described and we provide potential strategies for advancing the priority and note the proposed group that may guide the activity (ie, CDC, STLT public health departments, NGO partners).

### The 10 Essential Priorities

#### 1. Develop Threat-Specific Approaches

The aim of developing threat-specific approaches is to augment all-hazards planning, address the evolving threat environment, and support medical countermeasure logistics. This is accomplished by (1) using jurisdictional and national threat assessments to prioritize a threat-based planning approach and (2) developing guidance, tools, and resources for broad threat categories, including emerging infectious diseases and pandemics, chemical threats, environmental threats, radiological and nuclear threats, weather-related threats, and other threats.

In the past, efforts in this area have largely focused on anthrax and pandemic influenza planning. While these threats remain important to plan for, it is imperative that CDC incorporates individual jurisdictional risks into these planning efforts. Many jurisdictions have adopted this model already, and the framework calls for institutionalizing this approach. As part of this work, CDC, supported by NGO partners, will develop a threat-specific framework and a supporting suite of tools (including risk communications messages, fact sheets, case report forms, clinical guidance, guidance on the use of personal protective equipment, exercise scenarios) for each threat category. Similarly, the PHEP notice of funding opportunity will include requirements for STLT public health departments to develop response strategies to address primary threat categories identified in the jurisdiction’s risk assessment.

#### 2. Enhance Partnerships With Federal Agencies and Nongovernmental Organizations

The aim of enhancing partnerships with federal agencies and NGOs is to effectively support community preparedness. This is accomplished by (1) developing trusted partnerships across public health, emergency management, homeland security, healthcare sector, and community partners; (2) ensuring response-ready partnerships at all levels of government and community partners; and (3) enhancing coordination between public health and healthcare systems to improve readiness.

Lessons learned from COVID-19 and other recent public health emergencies indicate that pre-event partnerships are critical to ensure response readiness. As part of this new strategy, CDC will review and revise PHEP senior advisory committee members, as needed, to reflect new partnerships established during recent responses and reflect these new committee members in the upcoming notice of funding opportunity. CDC will work with interagency partners to reconvene an interagency grant and program coordination work group to ensure readiness-related collaboration and alignment among CDC, Administration for Strategic Preparedness and Response, Health Resources and Services Administration, Federal Emergency Management Agency, and other federal partners.

#### 3. Expand Local Support

The aim of expanding local support is to improve jurisdictional readiness to manage public health emergencies within local communities. This is accomplished by (1) enhancing local support by providing resources to local health departments, including large, highly populated cities and rural and frontier jurisdictions; and (2) expanding access to resources for local health departments, including directed funding, technical assistance, and human resources.

Responses typically start at the local level, and enhanced support is needed to best position local jurisdictions for successful public health emergency responses. As part of the framework strategy, CDC will be more proactive in setting targets for and assessing recipient support to local jurisdictions, including timely allocation of funding from states to local jurisdictions. CDC will also explore enhanced support for rural and frontier jurisdictions and work with NGO partners to develop realistic readiness and response capability expectations for these jurisdictions.

#### 4. Improve Administrative and Budget Preparedness Systems

The aim of improving administrative and budget preparedness systems is to support timely jurisdictional responses. This is accomplished by (1) ensuring jurisdictions continue to develop systems and processes to manage federal funds effectively and rapidly during a public health emergency and (2) prioritizing rapid hiring, procurement, and surge support.

Adequate and reliable administrative preparedness systems are critical for successful public health responses. CDC will place more focus on administrative readiness and plan to develop an additional public health preparedness and response capability standard focusing on administrative and budget preparedness. CDC, with the support of NGO partners, will also develop templates and guidance to assist STLT jurisdictions with administrative preparedness planning, financial risk mitigation, exercises, and training. In turn, CDC expects that STLT public health departments will update administrative preparedness plans to incorporate lessons learned from COVID-19 and other recent public health emergency responses.

#### 5. Build Workforce Capacity

The aim of building workforce capacity is to meet jurisdictional surge management needs and support staff recruitment, retention, resilience, and mental health. This is accomplished by (1) recruiting new and retaining qualified staff, enhancing training, and using creative solutions to address staffing shortages and (2) addressing the mental health needs and resiliency of the public health workforce.

An adequate number of trained, response-ready workers is essential for successful response operations. In direct support of the STLT workforce, CDC will continue to invest in the nationwide expansion of readiness and response field staff initiatives, including the Career Epidemiology Field Officer and Preparedness Field Assignee programs. To support these and other workers, CDC, in collaboration with NGOs and jurisdictional partners, will establish a community of practice that focuses on response outcomes, workforce resiliency, and emotional wellbeing.

#### 6. Modernize Data Collection and Systems

The aim of modernizing data collection and systems is to improve data quality and increase access to data, which promotes situational awareness and information sharing among healthcare and other partners during public health emergencies. This is accomplished by (1) identifying the critical data needs required from STLT jurisdictions and from CDC during a public health response and (2) establishing and enhancing systems for rapid data sharing to inform response operations and support decisionmaking.

The COVID-19 response illuminated the need for pre-event knowledge of essential data elements and pathways to transmit data to ensure critical situational awareness and information sharing across local, state, federal, and healthcare system response partners. As part of this new strategy, CDC, with the support of NGO partners, will work to better understand PHEP jurisdictions’ data modernization needs, goals, and progress and to identify the essential elements of information to support preparedness and response before an event. CDC will ask STLT public health departments to incorporate data and informatics needs into public health emergency response and continuity of operations plans.

#### 7. Strengthen Risk Communication Activities

The aim of strengthening risk communication activities is to improve the effectiveness of critical public health information and warnings. This is accomplished by (1) identifying strategies to combat misinformation and disinformation, (2) identifying strategies for regaining and retaining public trust, and (3) incorporating health equity practices to enhance preparedness and response support for communities experiencing differences in health status due to structural barriers.

While risk communication has long been a pillar of public health preparedness planning, the COVID-19 response identified a need for enhanced focus on this area, which is essential for advancing response readiness. As part of the next PHEP notice of funding opportunity, CDC will ask jurisdictions to update crisis and emergency risk communication plans to incorporate COVID-19 lessons learned and strategies to monitor and combat misinformation and disinformation. To support this work, CDC, in collaboration with NGO partners, will develop strategies for addressing misinformation and disinformation, identify promising practices across jurisdictions, and explore enhanced support for in-person and virtual crisis and emergency risk communication training.

#### 8. Incorporate Health Equity Practices

The aim of incorporating health equity practices is to enhance preparedness and response support for communities experiencing differences in health status due to structural and social barriers. This is accomplished by (1) ensuring STLT jurisdictions use health equity as a driver for preparedness planning, (2) identifying and mitigating factors that contribute to health disparities, and (3) supporting the hiring of health equity personnel.

Health equity has been a longstanding element of the PHEP program, but the new framework strategy calls for a more dedicated focus to incorporate health equity-related lessons learned into future planning efforts. CDC will update the 2024 PHEP notice of funding opportunity with current health equity standards and require that underserved populations have a seat at the planning table and are considered in planning and exercising. Specifically, CDC will direct recipients to identify disproportionately impacted populations, assess the risks to those populations, and develop strategies for mitigating those risks.

#### 9. Advance the Capacity and Capability of Public Health Laboratories

The aim of advancing the capacity and capability of public health laboratories is to rapidly characterize emerging threats through testing and surveillance. This is accomplished by investing in and modernizing jurisdictional public health laboratories to provide sufficient capacity and capability to rapidly identify and characterize biological and chemical threats and hazards.

As part of the framework, CDC and STLT public health departments will continue to support investments in Laboratory Response Network for Biological Threats and Laboratory Response Network for Chemical Threats and explore opportunities to support new and innovative public health laboratory initiatives. STLT public health departments will be required to incorporate laboratory operations into jurisdictional exercises, including exercising elements of laboratory surge.

#### 10. Prioritize Community Recovery Efforts

The aim of prioritizing community recovery efforts is to support health department reconstitution and resiliency. This can be accomplished by (1) supporting deliberate efforts to gather and use lessons learned from recent responses to strengthen programs and improve response readiness and (2) improving the resiliency of communities after public health threats and disasters.

Recovery is a critical and often overlooked phase of the disaster cycle but is critical to ensure communities are better positioned to respond to future public health emergencies.^1^ As part of the new framework, CDC will develop tools and templates for recovery-related plans and exercises and will work with NGO partners to align recovery resources, including grants, available to STLT public health departments across the interagency.

### Using the Framework to Create Action Plans

The Public Health Response Readiness Framework aligns with CDC’s overarching public health priorities and agency efforts to improve internal response readiness.^11^ DSLR convened 10 priority work groups to define framework activities and develop a 5-year action plan for each of the 10 areas that include proposed activities for DSLR’s PHEP program, the 62 PHEP jurisdictions, and partner organizations. DSLR is also designing an evaluation approach intended to measure success in implementing priority activities. A thorough exploration of each priority and related activities will be published in the 2024 PHEP notice of funding opportunity. Extensive supplemental guidance materials will be developed to help PHEP program recipients to meet program requirements. These materials can also aid organizations not funded by the PHEP program to examine their readiness and response plans. Creating a cohesive and coordinated strategy for improving response readiness will advance the PHEP program, maximize PHEP investments, direct the use of limited resources, and ensure CDC and its public health partners work together to reach common preparedness, response, and recovery goals.

## Looking Ahead

A modernized approach to public health readiness and response is essential to ensure federal and STLT public health entities leverage strengths, gaps, and lessons learned to improve future public health response operations. The CDC Public Health Response Readiness Framework is response informed and builds on progress made toward the 15 capabilities outlined *in Public Health Emergency Preparedness and Response Capabilities*.^3^ This new framework focused on response readiness offers a new tool for STLT public health preparedness practitioners and provides a roadmap for improved response operations. The framework priority areas are intended to guide PHEP program investments toward specific actions that improve response readiness for 21st century public health threats. The next generation of the PHEP program will better equip both CDC and STLT jurisdictions to protect and enhance the health and response readiness of communities nationwide.

## Figures and Tables

**Figure.**
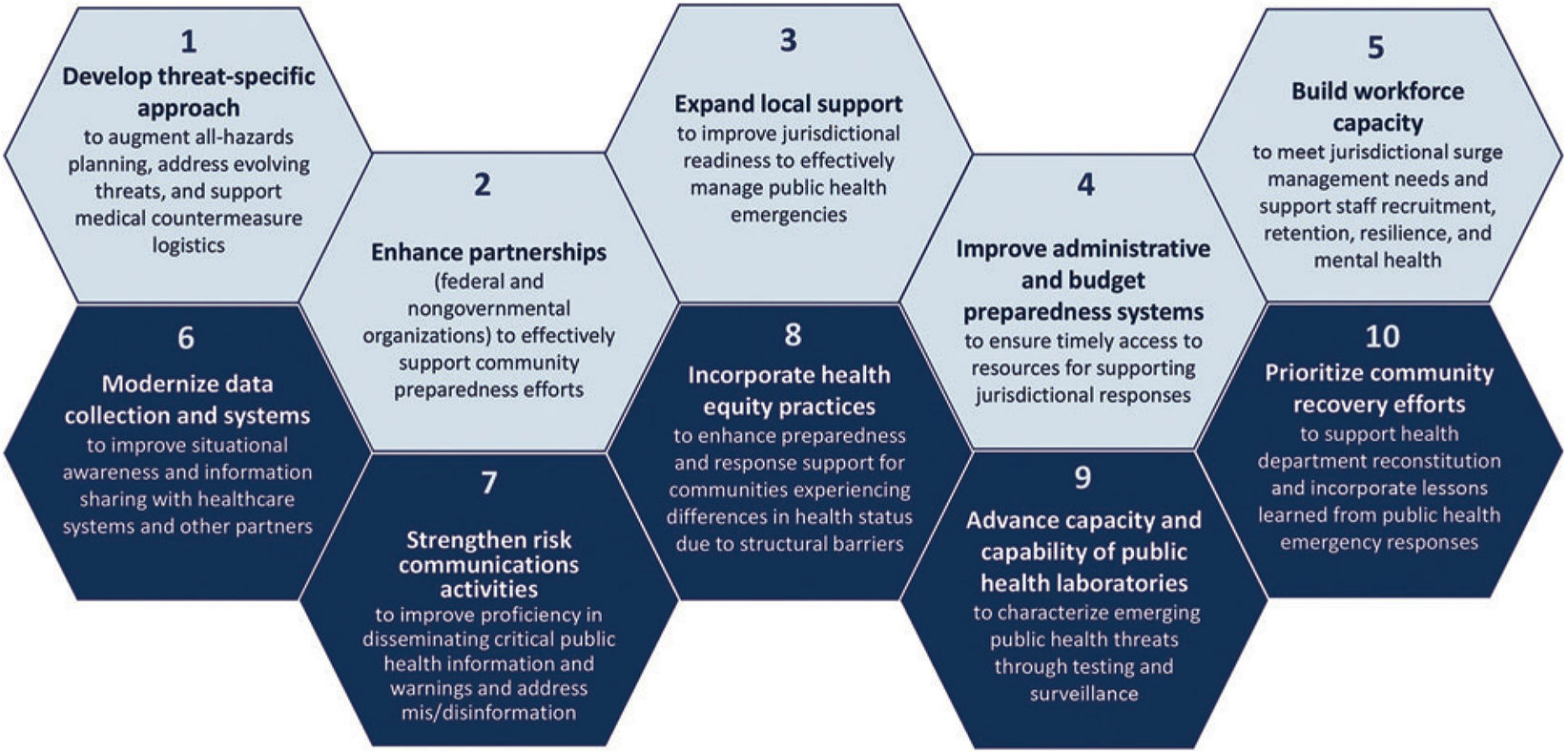
Public Health Response Readiness Framework. The fiscal years 2024-2028 PHEP program priorities that define excellence in response operations. Abbreviation: PHEP, Public Health Emergency Preparedness.

## Box. Key Sources of Input

- Next Generation of PHEP work groups
- ASTHO/NACCHO Evolution of PHEP project
- PHEP director meetings and calls
- ASTHO DPHP Executive Committee
- APHL, ASTHO, CSTE, NACCHO, PIHOA, and other partners
- CDC leaders who are former state or local health officials
- CDC and ORR strategic plans and priorities
- PHEP Operational Readiness Reviews
- 2022 DPHP annual meeting

## Abbreviations:

APHL: Association of Public Health Laboratories
ASTHO: Association of State and Territorial Health Officials
CDC: Centers for Disease Control and Prevention
CSTE: Council of State and Territorial Epidemiologists
DPHP: Directors of Public Health Preparedness
NACCHO: National Association of County and City Health Officials
ORR: Office of Readiness and Response
PHEP: Public Health Emergency Preparedness
PIHOA: Pacific Island Health Officers’ Association
